# Ready to work or not quite? Self-perception of practical skills among medical students from Serbia ahead of graduation

**DOI:** 10.3325/cmj.2015.56.375

**Published:** 2015-08

**Authors:** Tatjana Gazibara, Selmina Nurković, Gorica Marić, Ilma Kurtagić, Nikolina Kovačević, Darija Kisić-Tepavčević, Tatjana Pekmezović

**Affiliations:** Institute of Epidemiology, Faculty of Medicine, University of Belgrade, Belgrade, Serbia

## Abstract

**Aim:**

To assess final year medical students’ self-perception of their practical skills.

**Methods:**

The study was conducted at the Faculty of Medicine in Belgrade during compulsory practical sessions in the period December 2-9, 2013 and 390 students agreed to participate (response rate 77.8%). The questionnaire included questions on demographic characteristics, 21 questions on students' self-perception of their practical skills, and 1 question on students’ self-perceived readiness to start working with patients.

**Results:**

Cronbach’s α for the entire scale was 0.891. Students felt most confident about measuring arterial pulse and blood pressure and taking patients' history (average score 10 for all three skills) and least confident about placing a urinary catheter (average score 1) and suturing a wound (average score 2). They rated their readiness to work with patients with 5.0 out of 10.0 points. The total score did not correlate with students’ average mark (Spearman's ρ = 0.039; *P* = 0.460) and the average mark did not correlate with the self-perceived readiness to work with patients (Spearman's ρ = -0.048; *P* = 0.365).

**Conclusion:**

Our study suggests that medical students lack confidence to perform various clinical procedures, particularly those related to surgical interventions. To improve students’ confidence, clinical curriculum should include either more hours of practical work or ensure closer supervision of practical training in wards.

The ability to adequately perform patients’ examination, make a diagnosis, or prescribe appropriate therapy are crucial competencies that medical undergraduates have to attain (1). They are also indispensable for decreasing the incidence of adverse events in hospitals that are most commonly related to medical interventions and drug prescribing (2,3).

Recent studies have shown that clinical skills are most efficiently attained through implementation of technologies such as e-learning and video recorded simulations, and real time patient simulation (4-7). It has also been shown that students tend to underestimate their abilities and feel unconfident about performing the acquired skills (8). For example, observers and simulated patients rated medical students' skills better than the students themselves (9). Another study found that students did not significantly over- or underestimate their skills, but overestimated their ability to communicate with the patient (10). Additionally, students with low self-confidence and self-efficacy at performing certain clinical tasks were more likely to avoid these tasks in their daily work, while students with higher self-efficacy were more likely to persevere in difficult situations (11).

Medical education in Serbia has been entirely restructured since 2005 according to the Bologna Process principles (12). The aim of the reform was to improve medical curriculum and make students active participants in the patients’ treatment. Medical studies now last for 6 years (12 semesters) – first 3 years of preclinical and last 3 years of clinical training. Students start to work with patients in the clinical years, when they practice taking medical histories and performing clinical examinations under supervision of teaching assistants. When it comes to procedures such as wound suturing, blood sampling, placing of urinary catheters, cardio-pulmonary resuscitation, and patient immobilization, students mostly observe these procedures rather than performing them themselves. The reform aimed to create small-size teaching groups in which students would benefit from more interactions with teachers. Still, each year the Faculty admits a relatively high number of students (on average around 600 freshmen), which might influence the quality of teaching and learning.

Perception of clinical skills in an undergraduate setting should be evaluated in order to highlight potential weak points not only of individual students but of the training process as a whole. Therefore, the aim of this study was to evaluate the final year medical students’ perception of their own practical skills.

## Material and methods

### Participants

The questionnaire was distributed to 501 students of the sixth, final, year at the Faculty of Medicine, University of Belgrade, during compulsory practical sessions from December 2-9, 2013. 390 students agreed to participate (response rate 77.8%). Before the distribution of the questionnaires, four investigators described the purpose of the study and study procedures. Participation was anonymous and ethical approval was obtained from the Institutional Review Board of the Faculty of Medicine, University of Belgrade.

### Instrument

The questionnaire (English version in Supplementary material) was developed by our team. To check understanding and interpretation of items (in Serbian language), the questionnaire was validated in a pilot study on 20 medical students at the University of Belgrade. Several suggestions made by the students were incorporated into the final version.

The questionnaire collected the following demographic data: age, sex, average mark received for all six years. At oral exams at the University of Belgrade students are awarded from minimum 6 (at least 51 out of 100) to maximum 10 points (91-100 out of 100). The questionnaire also included 21 questions on students' self-perception of their patient management skills. The last question referred to self-perceived readiness to start working with patients. Answers were given on a scale from 1 to 10, where 1 meant “I am not confident about performing this skill at all,” and 10 meant “I am quite confident about performing this skill.” The total score was the sum of scores for all questions and ranged from 21 to 210.

### Data analysis

Internal consistency of the questionnaire was evaluated using Cronbach’s α coefficient (13). The suitability of data for the principal component analysis (PCA) was tested by Kaiser-Meyer-Olkin (KMO) measure of sampling adequacy and the Bartlett test of sphericity. After this, exploratory factor analysis (PCA with varimax rotation) was performed. Questionnaire subscales were obtained by grouping factor loadings. Factors with eigenvalues above 1.0 were considered important, as lower eigenvalues show that factor contributes little to the explanation of variances and may be left out. To explore the factor structure of the questionnaire, we determined factor clusters (subscales) based on the rotated component matrix: factor loadings (ie, correlation coefficients between the scale items and established factors) were grouped according to related values starting from the highest to the lowest value in order to cover a coherent cluster of questionnaire items. Finally, each factor cluster was named according to common features related to selected items (skills).

Kolmogorov-Smirnov test showed not-normal distribution of practical skills ratings. Therefore, the data are presented as medians and interquartile ranges. Differences in scores between sexes were assessed by Mann-Whitney U test for two independent samples. Spearman’s correlation test was used to investigate the correlations between average mark and total skill score and as well as level of self-perceived readiness to start to work with patients. *P* < 0.05 was considered significant. Statistical analysis was performed using SPSS 17.0 (SPSS Inc, Chicago, IL, USA).

## Results

### Questionnaire structure

The Cronbach’s alpha coefficient for the entire scale was 0.891. Sampling adequacy according to the KMO criteria was excellent (0.874) and Bartlett test of sphericity yielded a probability value of *P* < 0.001. Factor analysis reduced the number of 21 items in the questionnaire to 5 factors with eigenvalue above 1.0 (Table 1). Eigenvalues for the 5 factors were 7.01, 2.31, 1.58, 1.32, and 1.04. Four skills were grouped in Factors 1, 3, and 4, and five skills were grouped in Factors 2 and 5. Each cluster was named according to skills it contained: 1 – “Major interventions (physically demanding);” 2 – “Minor interventions (fine manual skills);” 3 – “Results interpretation;” 4 – “Basic patient assessment;” and 5 – “Other skills.” The highest factorial loadings for each factor were the following: Factor 1 – performing cardiopulmonary resuscitation (0.850), Factor 2 – taking venous blood sample (0.718), Factor 3 – interpreting ECG (0.812), Factor 4 – arterial pulse measurement (0.742), Factor 5 – reflex examining (0.650). The total matrix variance for all factors was 60.3% (for Factor 1 – 31.9%, Factor 2 – 10.5%, Factor 3 – 7.2%, Factor 4 – 6.0%, and for Factor 5 – 6.7%).

**Table 1:** Principal component analysis (with varimax rotation) of the questionnaire*

Item	Skills	Factorial load				
		Factor 1 major interventions (physically demanding)	Factor 2 minor interventions (fine manual skills)	Factor 3 results interpretation	Factor 4 basic patient assessment	Factor 5 other skills
1	Taking patients' history	-0.056	0.092	0.192	**0.680**	0.146
2	Performing physical examination	-0.098	0.291	0.366	**0.554**	0.246
3	Differentiation of heart sounds	0.065	0.338	**0.593**	0.245	0.028
4	Arterial pulse measurement	0.143	-0.085	0.075	**0.742**	0.039
5	Blood pressure measurement	0.152	0.112	0.020	**0.639**	-.0.001
6	Taking venous blood sample	0.298	**0.718**	0.076	0.157	-0.085
7	Suturing a wound	0.202	**0.704**	0.108	-0.037	0.208
8	Wound bandaging	0.525	**0.519**	0.137	0.286	0.081
9	Performing the Heimlich maneuver	**0.641**	0.357	0.088	0.047	0.239
10	Administering intramuscular injection	0.537	**0.493**	0.002	0.289	-0.064
11	Performing cardiopulmonary resuscitation	**0.850**	0.104	0.214	-0.022	0.114
12	Immobilizing a patient	**0.800**	0.227	0.179	0.036	0.222
13	Wound management	**0.657**	0.386	0.195	0.181	0.146
14	Interpreting a RTG	0.144	0.128	**0.748**	0.026	0.117
15	Interpreting an ECG	0.106	0.081	**0.812**	0.095	0.017
16	Interpretation of blood test	0.225	-0.021	**0.725**	0.259	0.056
17	Placing an urinary catheter	0.213	**0.681**	0.198	-0.018	0.038
18	Throat examination	0.196	-0.031	0.215	0.329	**0.518**
19	Reflexes examination	0.076	-0.176	0.122	0.354	**0.650**
20	Digital rectal examination	0.195	0.270	-0.158	-0.004	**0.633**
21	Exploring evidence-based medicine data	0.156	0.249	0.375	-0.171	**0.538**
22	Readiness to start working with patients	0.217	0.475	0.451	0.051	**0.328**

*Bold values denote eigenvalues for the factors.

### Response analysis

Of 390 medical students, 34.4% (135) were men. Median age of participants was 24 years (IQR, 23-37). Students felt most confident about measuring arterial pulse and blood pressure and taking patients' history (Table 2) and least confident about placing a urinary catheter and suturing a wound (Table 2). Female students were significantly more confident about taking patients' history (Z = -5.624; *P* = 0.001) and performing physical examination (Z = -2.541; *P* = 0.011) than male students. Male students, however, felt significantly more confident about suturing a wound (Z = -2.449; *P* = 0.014), performing the Heimlich maneuver (Z = -3.415; *P* = 0.001), performing cardiopulmonary resuscitation (Z = -3.092; *P* = 0.002), and exploring evidence-based medicine data (Z = -2.896; *P* = 0.004). They also felt more ready to start working with patients (Z = -2.180; *P* = 0.029). In terms of subscales scores, female students were significantly more confident about performing basic patient assessment (Factor 4) than male students (Table 2).

**Table 2:** Average scores of practical skills perceived by final-year medical students according to questionnaire domains and sex*^†^

Factor^‡^	Domains	All N = 390	Men N = 135	Women N = 255	*P* for sex difference
1	**Major interventions (physically demanding)** Cronbach's α = 0.867				
	Performing the Heimlich maneuver	5.0 (7.0)	6.0 (5.0)	3.0 (6.0)	0.001
	Performing cardiopulmonary resuscitation	5.0 (5.0)	6.0 (4.7)	5.0 (5.0)	0.002
	Immobilizing a patient	5.0 (5.0)	5.0 (4.0)	4.0 (6.0)	0.077
	Wound management	6.0 (5.0)	6.0 (5.0)	5.0 (5.0)	0.500
	Subscale score	20.0 (18.0)	22.0 (18.0)	19.0 (18.0)	0.071
2	**Minor interventions (fine manual involvement)** Cronbach's α = 0.812				
	Taking venous blood sample	3.5 (7.0)	4.0 (6.0)	3.0 (7.0)	0.294
	Suturing a wound	2.0 (5.0)	3.0 (6.0)	2.0 (4.0)	0.014
	Wound bandaging	6.0 (5.0)	6.0 (4.0)	7.0 (6.0)	0.548
	Administering intramuscular injection	7.0 (8.0)	7.0 (7.0)	7.0 (8.0)	0.915
	Placing an urinary catheter	1.0 (4.0)	1.0 (5.0)	1.0 (4.0)	0.455
	Subscale score	21.0 (21.0)	23.0 (20.2)	21.0 (20.0)	0.511
3	**Results interpretation** Cronbach's α = 0.780				
	Differentiation of heart sounds	7.0 (3.0)	7.0 (3.0)	6.0 (3.0)	0.701
	Interpreting a RTG	6.0 (3.0)	7.0 (3.0)	6.0 (4.0)	0.203
	Interpreting an ECG	7.0 (3.0)	6.0 (3.0)	7.0 (3.0)	0.653
	Interpretation of blood test	8.0 (3.0)	8.0 (3.0)	8.0 (3.0)	0.146
	Subscale score	27.0 (11.0)	27.0 (12.0)	27.0 (10.0)	0.821
4	**Basic patient assessment** Cronbach's α = 0.676				
	Taking patient's history	10.0 (1.0)	9.0 (2.0)	10.0 (1.0)	0.001
	Performing physical examination	9.0 (1.0)	8.0 (2.0)	9.0 (2.0)	0.011
	Arterial pulse measurement	10.0 (1.0)	10.0 (1.0)	10.0 (1.0)	0.086
	Blood pressure measurement	10.0 (1.0)	10.0 (1.0)	10.0 (1.0)	0.150
	Subscale score	37.0 (5.0)	36.0 (6.2)	37.0 (4.0)	0.010
5	**Other skills** Cronbach's α = 0.613				
	Throat examination	8.0 (3.0)	8.0 (3.7)	8.0 (3.0)	0.099
	Reflexes examination	9.0 (2.0)	9.0 (3.0)	9.0 (2.0)	0.079
	Digital rectal examination	5.0 (8.0)	6.0 (6.7)	5.0 (8.0)	0.122
	Exploring evidence-based medicine data	5.0 (6.0)	7.0 (5.0)	5.0 (6.0)	0.004
	Readiness to start working with patients	5.0 (5.0)	6.0 (4.0)	5.0 (4.0)	0.029
	Subscale score	31.0 (14.0)	33.0 (13.0)	30.0 (13.7)	0.163

*medians and interquartile ranges.

†Scale responses: 1 – I am not able to perform the given skill at all; 10 – I am confident about performing the given skill.

‡Score range for Factors 1, 3, and 4 was 4-40; score range for Factors 2 and 5 was 5-50.

**Figure 1:**
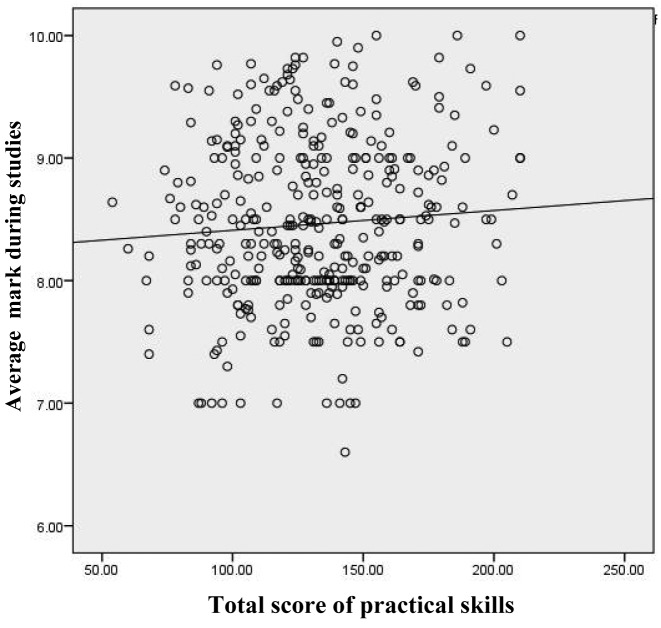
Correlation between the total score of practical skills and the average mark received for all 6 years of studies.

**Figure 2:**
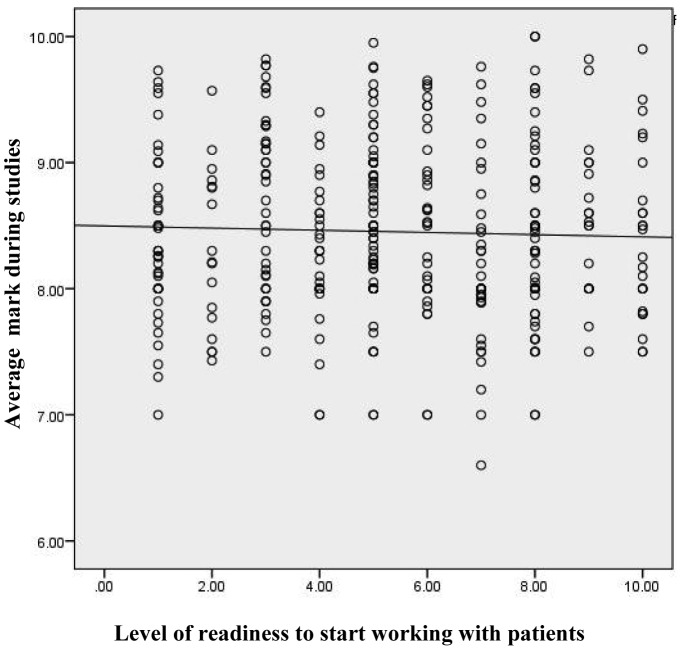
Correlation between average mark received for all 6 years of studies and the level of readiness to start working with patients.

The total skill score did not correlate with average mark (Spearman's ρ = 0.039; *P* = 0.460) (Figure 1) and the average mark did not correlate with the level of self-perceived readiness to start working with patients (Spearman's ρ = -0.048; *P* = 0.365) (Figure 2).

## Discussion

In our study, students felt most confident about measuring arterial pulse and blood pressure and taking patients' history and least confident about placing a urinary catheter and suturing a wound. Additionally, the total score of skills did not correlate with students’ average mark, and the average mark did not correlate with the self-perceived readiness to work with patients.

The finding that students felt most confident about taking history and measuring arterial pulse and blood pressure could be explained by the fact that these skills students start practicing first and they are practiced consistently throughout clinical undergraduate training. Also, arterial pulse measurement is a simple procedure that does not require any particular instrument and it even can be practiced without an actual patient. Although studies have shown that medical students are not familiar with theoretical guidelines on blood pressure measurement (14,15), our students felt quite confident about performing this procedure. They also felt rather confident about performing physical examination, although this skill did not reach the highest score. Potential reason for this could be that not every student has an opportunity to practice examination due to a large number of students and limited number of teaching staff. A study from Brazil also found that students were most confident about performing physical examination (16). Our students felt least confident about placing urinary catheters and suturing wounds. This is not surprising since these procedures are not routinely performed during undergraduate medical training. The majority of other skills scores got medium ratings, suggesting that although students did not feel overly confident, they were familiar with these procedures. This finding can be explained by the fact that students observed the performing of these procedures but did not perform them themselves.

Medical students in Croatia, which also implemented the Bologna reform in 2005, expressed higher self-confidence about performing overall practical skills and patient management (17), but lower confidence about performing basic surgical skills and placement of urinary catheters, which is quite similar to our findings (17). In contrast, North American medical students felt most confident about inserting urinary catheter (18). Such divergent findings could be attributed to the fact that in the US students are actually involved in patient treatment by being on-call and working in in- and out-patient departments, while in Serbia this is not part of obligatory medical curriculum. Our students are free, but not encouraged, to take part in night shifts if approved by their clinical supervisor/teacher. Nonetheless, students in the USA, Brazil, and Croatia perceived lack of confidence about performing basic surgical procedures (16-18). To improve surgical skills students require actual circumstances in which they could practice and build their confidence, such as weekend workshops (19).

This study observed similar sex differences as other studies in the type of skill that student felt most confident about (20-23). For example, a study from Croatia indicated that men performed significantly more practical medical procedures than women (20). Also, a study in the UK reported that women performed clinical examination better than men (21), which is in accordance with our results. Such difference between sexes could have resulted from the fact that women study more and therefore are more prepared (24), but are also motivated by humanist and altruistic reasons (23). Male students, on the other hand, usually prefer surgical specialties when it comes to career choices, so it is not surprising that they feel more confident about performing surgical skills (22,23).

There was a stunning discrepancy between self-perception of practical skills and the average marks. Although students tend to underestimate their patient management skills (9,25), in our study students with excellent marks did not have corresponding self-confidence in their skills. It is possible that students with higher marks are more self-critical and therefore rate their skills lower. Likewise, the average mark does not necessarily reflect students' practical performance. In Serbia the final marks are received after oral examination, scheduled after the practical part of the exam with patient examination, and may depend on the subjective impression of the teacher.

Similarly, we observed a striking discrepancy between the average mark and the level of readiness to start working with patients. Overall, students' readiness was not encouraging, which suggests that they lacked actual, real-time practice. However, it may also suggest that the hours students spent in wards were not effectively dedicated to development of skills either because the students are not officially required to perform a certain number of procedures to pass the final exam or because they lack self-initiative. To improve students’ confidence, it is indispensible that students are involved in all procedures carried out at that particular department.

Although our sample size was considerably large, the questionnaire used in the study referred to basic practical skills only. A limitation of the study could be participation bias and the questionnaire could have benefitted from inclusion of other dimensions such as understanding and interpreting scientific results, prevention of communicable and non-communicable diseases, and interpersonal and coping skills. All these domains should be evaluated in future research using more extensive and comprehensive questionnaires. Also, students' actual practical skills should be evaluated after completion of the questionnaire by independent observers. Although the questionnaire had overall good psychometric properties, we observed that some subscales (“Basic patient assessment” and “Other skills”) had somewhat lower Cronbach's α. Some authors have suggested that the α coefficient can be lower if the scales have fewer than 10 items, but still the they can have sufficient validity and there is justified theoretical and practical reasoning for the inclusion of items (26). Given the validity of the questionnaire, we propose that it is translated and validated in other languages. Finally, we did not assess students' skills before the implementation of the Bologna reform and therefore we could not make a pre- and post comparison.

In conclusion, our study suggests that medical students lack confidence about performing various clinical procedures, particularly those related to surgical interventions. To improve students’ confidence, clinical curriculum should include either more hours of practical work or ensure adequate supervision of students’ practical work. Introduction of skill self-assessment logbooks may help students to improve their clinical performance and increase confidence before completion of undergraduate medical training.
